# Accelerating 3D radial MPnRAGE using a self‐supervised deep factor model

**DOI:** 10.1002/mrm.30549

**Published:** 2025-06-02

**Authors:** Yan Chen, Steve R. Kecskemeti, James H. Holmes, Curtis A. Corum, Nima Yaghoobi, Vincent A. Magnotta, Mathews Jacob

**Affiliations:** ^1^ Electrical and Computer Engineering University of Virginia Charlottesville Virginia USA; ^2^ Waisman Center University of Wisconsin‐Madison Madison Wisconsin USA; ^3^ Department of Radiology University of Iowa Iowa City Iowa USA; ^4^ Biomedical Engineering and Hawk‐IDDRC University of Iowa Iowa City Iowa USA; ^5^ Champaign Imaging LLC Shoreview Minnesota USA; ^6^ Department of Psychiatry University of Iowa Iowa City Iowa USA

**Keywords:** MPnRAGE, multi‐contrast MRI, quantitative MRI, single‐shot reconstruction

## Abstract

**Purpose:**

To develop a self‐supervised and memory‐efficient deep learning image reconstruction method for 4D non‐Cartesian MRI with high resolution and a large parametric dimension.

**Methods:**

The deep factor model (DFM) represents a parametric series of 3D multicontrast images using a neural network conditioned by the inversion time using efficient zero‐filled reconstructions as input estimates. The model parameters are learned in a single‐shot learning (SSL) fashion from the k‐space data of each acquisition. A compatible transfer learning (TL) approach using previously acquired data is also developed to reduce reconstruction time. The DFM is compared to subspace methods with different regularization strategies in a series of phantom and in vivo experiments using the MPnRAGE acquisition for multicontrast $T_{1}$ imaging and quantitative $T_{1}$ estimation.

**Results:**

DFM‐SSL improved the image quality and reduced bias and variance in quantitative $T_{1}$ estimates in both phantom and in vivo studies, outperforming all other tested methods. DFM‐TL reduced the inference time while maintaining a performance comparable to DFM‐SSL and outperforming subspace methods with multiple regularization techniques.

**Conclusion:**

The proposed DFM offers a superior representation of the multicontrast images compared to subspace models, especially in the highly accelerated MPnRAGE setting. The self‐supervised training is ideal for methods with both high resolution and a large parametric dimension, where training neural networks can become computationally demanding without a dedicated high‐end GPU array.

## 1 INTRODUCTION

Inversion recovery (IR) methods are often used in neurological imaging to obtain optimal tissue contrasts for qualitative visual assessment as well as to derive quantitative $T_{1}$ maps.^1^ The parameters of the IR sequence are usually optimized for typical tissue $T_{1}$ values.^2^, ^3^ However, this method becomes challenging when applied to pediatric and aging populations due to the variability in $T_{1}$ caused by (de‐)myelination; the inversion time must be tuned according to the subject to provide optimal contrast. In addition, the delays for inversion and magnetization recovery result in longer acquisition times in high‐resolution settings. MPnRAGE^4^ is an extension of the widely used MPRAGE^5^ and MP2RAGE,^6^ in which only one or two $T_{1}$‐weighted images are acquired after the inversion pulse. In contrast, MPnRAGE acquires a large number (N) of gradient echo radial readouts within each inversion block using pseudo‐random view ordering.^4^ Although each of the N images along the inversion recovery curve is highly undersampled, there are correlations across the images that can potentially be exploited using constrained image reconstruction algorithms.

The challenges of high‐dimensional image reconstruction from MPnRAGE, for example, 3D + time, are the high memory demand and computational load, especially when N is on the order of hundreds. The low‐rank property has been extensively explored based on spatial and temporal correlations.^7^, ^8^ Globally low‐rank (GLR) methods approximate the fully sampled high‐dimensional image series by a lower‐dimensional matrix. In the undersampled setting, missing matrix entries are completed using nuclear norm minimization. GLR has been applied to dynamic MRI^9^, ^10^, ^11^, ^12^ and MR fingerprinting.^13^, ^14^ Locally low‐rank (LLR) methods divide the entire image into a partition of patches and minimize the rank of submatrices for each patch.^15^, ^16^ While LLR offers better performance than GLR, its high computational complexity often limits its use in 3D + time problems. A closely related class is subspace methods, which explicitly decompose the full image series into temporal basis functions and spatial coefficients.^17^ The temporal basis functions are typically predetermined from the Bloch simulations. This predetermination greatly reduces the degrees of freedom and eliminates the need for nuclear norm computation, thus being more computationally and memory efficient compared to GLR.^18^ Additional regularization in the spatial domain is desirable to reduce aliasing artifacts resulting from subsampling, for example, wavelet $\ell_{1}$ norm regularization^19^ and total variation priors.^20^

In recent years, deep learning methods have been explored to improve constrained factorization methods. A deep Hankel matrix factorization network (DHMF) learns the low‐rank prior to avoid the time‐consuming singular value decomposition (SVD) in the recovery of exponential signals.^21^ The model‐based supervised subspace learning approach^22^, ^23^ is based on a convolutional neural network (CNN) regularizer on spatial coefficients, where the CNN parameters are learned in an end‐to‐end (E2E) fashion. Although E2E training offers good performance, it requires a large training dataset consisting of fully sampled volumes,^24^, ^25^ which are not available in the 3D + time setting. Model‐based self‐supervised approaches^26^, ^27^ eliminate the need for ground truth images by training a CNN using undersampled k‐space data, but they are difficult to apply to 3D acquisitions due to the memory demand of unrolling. Hence, current methods rely on Cartesian sampling, where each slice is independently solved using a slice‐by‐slice^28^ approach or by slabs^29^; however, direct extension to our 3D radial setting remains challenging. A memory‐efficient unrolling method that trades computation for memory has been introduced^30^, ^31^; however, it has not yet been applied to 3D + time reconstruction, likely due to its high computational demands.

In this work, we introduce a self‐supervised single‐shot approach for the recovery of multicontrast MPnRAGE data. Unlike supervised deep learning methods, the proposed SSL approach does not require training data. Instead, the network parameters are learned from the undersampled k‐t space data in a manner similar to Deep Image Prior (DIP),^32^ which is more memory‐efficient than unrolling of model‐based methods.^26^, ^33^ This approach benefits from the bias of CNNs toward images,^32^ which offers spatial regularization.

Unlike DIP initializes the network with random noise, the proposed method uses zero‐filled images as inputs, which can be quickly and efficiently reconstructed using NUFFT, inspired by the DEBLUR approach.^34^ This approach enables the use of the learned model weights from one or multiple datasets as initialization for a new dataset, a technique known as transfer learning (TL). Fewer adaptation steps are often sufficient to recover a new dataset, resulting in reduced inference time over fully self‐supervised learning.

The proposed self‐supervised model has conceptual similarities to implicit neural representations (INRs), which represent the signal as a continuous mapping from coordinates.^35^, ^36^, ^37^, ^38^ Although this approach has been successfully demonstrated in 2D settings, it is not practical in our 3D + time application. In particular, the storage of large multidimensional volumes at each layer of the coordinate network leads to a high memory demand. In addition, the INR approach does not allow for the reuse of reconstruction weights from one dataset to another, which is possible with our approach.

## 2 METHODS

### 2.1 Background

#### 2.1.1 Forward model

The forward model

(1) $$y_{\tau }={𝒜}_{\tau }\eta_{\tau }+n_{\tau }$$

is used to relate the measured k‐space signal $y_{\tau }$ to a set of unknown images $\eta =[\eta_{1},\eta_{2},\eta_{3},\ldots ,\eta_{T}]$ and measurement noise $n_{\tau }$. ${𝒜}_{\tau }$ denotes the forward model at time τ for each excitation.

#### 2.1.2 Subspace reconstruction

The rank of the Casorati matrix η is generally much lower than the total number of distinct inversion times (TI).^8^, ^17^ The subspace approach models the Casorati matrix as the product of two‐factor matrices, the spatial factor U and the temporal factor V:

(2) η=UV

The columns of U are spatial coefficient images, while the columns of V are temporal basis functions. The low‐rank factor model in (2) can be alternatively written as

(3) η(τ)=UV(τ),

where τ denotes TI.

#### 2.1.3 Deep image prior

Deep image prior (DIP) represents the clean image as the output of a network ${𝒩}_{\theta }(z)$ with weights θ, where z is often chosen as white noise, represented as follows for MRI reconstruction:

(4) $$\theta^{*}=\arg\underset{\theta }{\min}\|𝒜({𝒩}_{\theta }(z))-y{\|}^{2}.$$

where θ are optimized such that $𝒜({𝒩}_{\theta }(z))$ matches the measurements y.

### 2.2 Deep factor model

**FIGURE 1:**
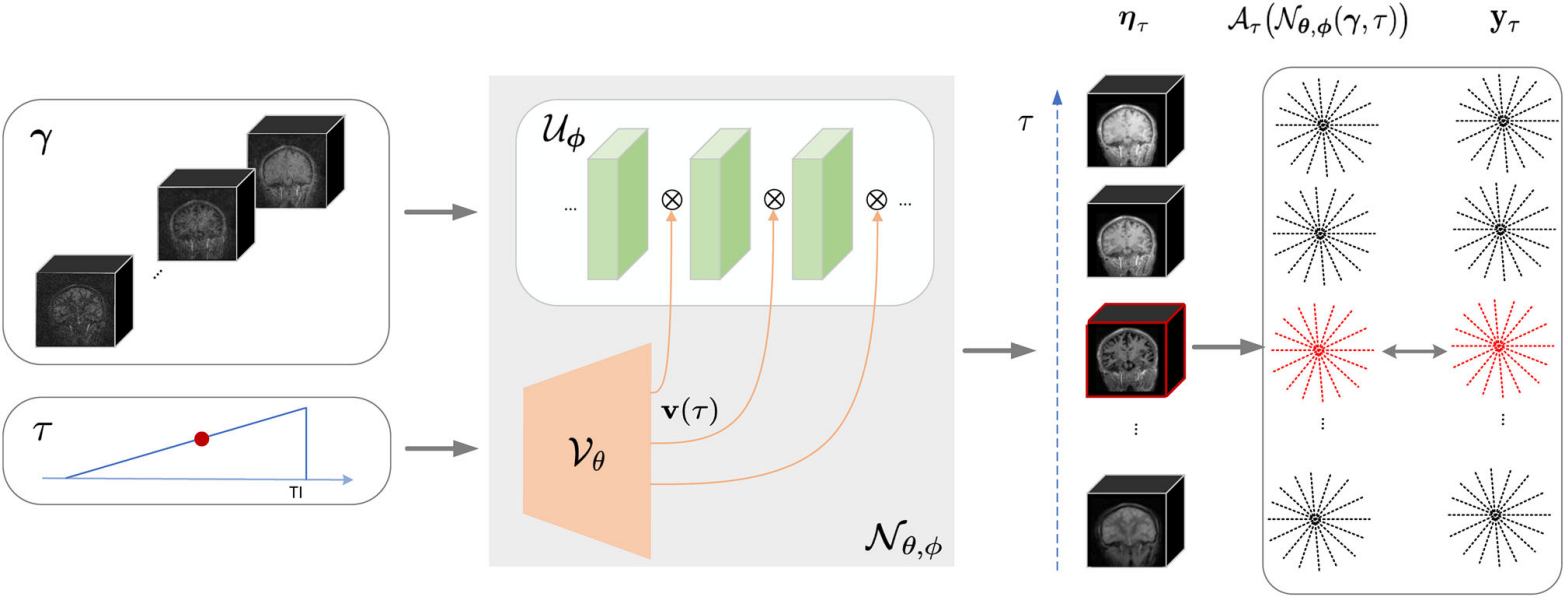
Illustration of the deep factor model for the recovery of multicontrast 3D radial MPnRAGE data. DFM comprises a CNN indicated by ${𝒰}_{\phi }$ and a fully connected MLP ${𝒱}_{\theta }$. The input to ${𝒰}_{\phi }$ is the 3D coarse reconstruction γ, which consists of eight volumes obtained by gridding eight subsets of k‐space data, grouped according to the inversion time(TI). ${𝒱}_{\theta }$ modulates the CNN features by learning the nonlinear function v(τ) of the inversion time condition τ, which is linearly sampled between 0 and 1 with 384 steps representing 384 contrasts generated at each RF pulse. Channelwise multiplication is applied between v(τ) and the intermediate CNN features. The parameters ϕ and θ are jointly learned using a loss between the forward model and the acquired k‐space data for each contrast. Using the zero‐filled input γ instead of white noise enables the learned parameters from one subject to be easily transferred to another. This accelerates training by offering a superior initialization compared to random initialization.

The image $\eta_{\tau }$ at a specific inversion time τ is modeled as the output of a deep conditional network ${𝒩}_{\theta ,\phi }$ as shown in Figure 1. The model ${𝒩}_{\theta ,\phi }$ consists of a CNN ${𝒰}_{\phi }$ and a dense network ${𝒱}_{\theta }$. The feature maps in each CNN layer of ${𝒰}_{\phi }$ are modulated by temporal factors v(τ) using channel‐wise multiplication. v(τ) is derived from the fully connected network ${𝒱}_{\theta }$:

(5) $$v(\tau )={𝒱}_{\theta }(\tau ).$$

Thus, the image at TI τ is as the following:

(6) $$\eta (\tau )={𝒰}_{\phi }\left(\gamma ,v(\tau )\right)={𝒩}_{\theta ,\phi }(\gamma ,\tau ).$$

where γ is a zero‐filled image reconstructed from eight uniform bins based on the inversion time. γ may be corrupted by extensive aliasing artifacts resulting from undersampling. ${𝒩}_{\theta ,\phi }$ may thus be seen as a denoising function, where τ is a condition vector to account for the inversion‐time‐dependent contrast.

θ and ϕ are optimized as follows:

(7) $$\{\theta^{*},\phi^{*}\}=\arg\underset{\theta ,\phi }{\min}\;\underset{\tau }{\sum }\|{𝒜}_{\tau }({𝒩}_{\theta ,\phi }(\gamma ,\tau ))-y_{\tau }{\|}^{2}.$$

θ and ϕ are learned in a self‐supervised fashion using the available k‐space measurements. The proposed approach has some similarities to factor‐based models (e.g., subspace methods). In particular, if the number of CNN layers is one, $\eta_{\tau }=Uv(\tau )$, where U denotes the CNN features. In this case, the proposed approach is similar to DEBLUR.^34^

### 2.3 Implementation details

#### 2.3.1 Data acquisition and post‐processing

##### Pulse sequence

MPnRAGE^4^ is a 3D radial inversion recovery sequence. The base sequence consists of an adiabatic inversion pulse followed by gradient echoes. Please see Figure S9 in Supporting Information for the pulse sequence. The MPnRAGE scan was performed with FOV = 256×256×256 mm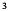, resolution = 1×1×1 mm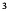, TR = 4.88 ms, duration of inversion pulse = 12 ms. Three hundred and eighty‐five radial readouts per inversion block were collected. The readout from the first RF pulse was excluded because of incomplete spoiling after the inversion pulse. The images of 384 TIs were reconstructed. After the gradient echoes, a delay time $T_{D}=503.5$ ms allows the longitudinal magnetization to freely regrow before the next inversion pulse. The k‐space view ordering was arranged in a pseudorandom ordering so that the 385 views collected after each inversion block approximately uniformly sample the edges of k‐space. Likewise, the views from each successive inversion block fill in the gaps to satisfy two conditions: (1) the cumulative sum of all collected views approximately uniformly samples k‐space, and (2) the cumulative sum across inversion blocks for each view, that is, inversion time, approximately uniformly samples k‐space. Retrospective subsampling can then be achieved by using only a subset of views from consecutive inversion blocks.^39^

Radial readouts with golden‐angle view ordering^4^ were considered. To facilitate the estimation of $B_{1}$ inhomogeneity and inversion efficiency, a variable flip angle approach was used in each inversion block, where the first 304 gradient echoes were acquired using a $4^{\circ }$ flip angle and the last 81 RF pulses with $8^{\circ }$. In this work, the complete dataset was acquired with 224 inversion blocks in 9 min, which was sufficient for $T_{1}$ mapping by nonlinear fitting of Bloch models using zero‐filled estimation of images. The dataset was retrospectively subsampled by only retaining a subset of inversion blocks. For example, retaining the first 56 inversion blocks translated to a 2.3 min scan. Subsets with durations of 4.5, 2.3, and 1.1 min were considered. The results of the 4.5 and 1.1 min scans are shown in Supporting Information Figures S1, S2, S4, and S5.

##### Phantom data

The High Precision Devices system phantom (model 130; Boulder, CO) that provided traceability to NIST measurements was imaged^40^ on a clinical 3T scanner (Signa™ Premier, GE Healthcare, Waukesha, WI) using a 48‐channel receive‐only RF head coil. The original 48 channels of data were compressed to 4 virtual coils using principal component analysis (PCA).^41^ The coil sensitivity maps of the virtual coils were estimated using the JSENSE^42^ method.

To assess the accuracy of $T_{1}$ estimates, we chose six vials whose $T_{1}$ values were between 300 and 2000 ms. A circular mask with a radius of 6 mm was drawn to extract the voxels within each vial.

##### In vivo data

Six in vivo datasets were acquired from six healthy adults (2 women and 4 men, 20–50 years of age) using an IRB‐approved protocol and receiving informed written consent. Participants were instructed to remain still. Data acquisition and processing methods matched the phantom data acquisition.

### 2.4 Neural network architecture

If the input to the DIP network is white noise, DIP requires a large number of parameters in CNN to converge to the true solution. Inspired by DEBLUR,^34^ we used an informative input (approximate solution) instead of white noise to reduce the number of parameters. ${𝒰}_{\phi }$ (Equation 6) was implemented as a 3D neural network with three 3D CNN blocks (Figure 1). The first two blocks each contained a 3D CNN layer, a channelwise multiplication layer modulated by temporal factors v(τ) from ${𝒱}_{\theta }$, and a nonlinear activation layer. Each CNN layer had a 3×3×3 kernel, a stride of 1, and no padding. The first block processed 16 input channels, including the real and imaginary components of eight volumes of γ. The number of layers and channels followed the ablation study (Supporting Information Figure S7). The outputs were two feature channels representing the complex image. The first CNN block used a Tanh activation, while the others applied Leaky‐ReLU with a 0.2 negative slope.

${𝒱}_{\theta }$ was implemented as 3 linear layers using 2D CNN with a kernel size of 1×1, a stride of 1, and no padding, followed by a Leaky‐ReLU function with a 0.1 negative slope.

### 2.5 Training of the model

#### 2.5.1 Single‐shot learning

The input to the CNN was the undersampled gridding reconstruction γ. γ denotes eight 3D volumes, each obtained using iNUFFT for one of eight k‐space data subsets binned based on TI.^43^, ^44^

Training on images from 384 TIs was computationally intensive, requiring 384 forward and backward passes per optimization step with SGD. To accelerate this, we adopted an annealing approach in k‐space. The 384 TIs were initially grouped into 32 subgroups based on TI, and the network was trained with τ varying in 32 steps. After convergence, the number of subgroups was gradually increased to 384.

A progressive‐in‐space strategy was utilized to manage memory usage. Training started with a matrix size of $128^{3}$; then the learned weights were used to initialize training at $256^{3}$. The $128^{3}$ training required approximately 10 GB of memory on an NVIDIA A100 GPU, while $256^{3}$ needed 74 GB.

Progressive training was implemented in five stages with varying matrix sizes and temporal resolutions: matrix sizes $\left[128^{3},128^{3},128^{3},256^{3},256^{3}\right]$ and temporal subgroups [32,64,384,64,384]. The initial learning rates were set to $\left[1\times 10^{-3},5\times 10^{-4},5\times 10^{-4},5\times 10^{-4},5\times 10^{-4}\right]$.

The first four stages accelerated training using fixed epochs and learning rates: [30,30,60,20] epochs, with learning rate decay at epochs [20,20,40,10] (decay rate = 0.2). In the final stage, a reduce‐on‐plateau scheduler and early stopping were applied to mitigate overfitting (Supporting Information Figure S8). The runtime per epoch ranged from 0.8 min (first stage: 32 subgroups, $128^{3}$) to 12.4 min (final stage: 384 subgroups, $256^{3}$).

Adam optimizer was applied to optimize the network using default hyperparameters within PyTorch.^45^ The k‐space measurements were normalized so that the maximum intensity of the composite gridded image was one.

#### 2.5.2 Transfer learning

A key difference between the proposed scheme and DIP is the use of the gridding reconstruction γ as input to the network. The anatomical information of the specific subject is captured by γ; the network parameters that *denoise* γ are more generic across subjects. This approach allows us to use the model weights learned from one or multiple subjects to initialize a new subject.

Directly applying a DFM model trained on one subject to another does not guarantee data consistency. Instead, transfer learning was employed. A DFM was pretrained using single‐shot learning (Section 2.5.1) on one subject, and then the learned weights served to initialize for another subject. The subsampling rates of input γ remained consistent with the pretrained model, and training followed the final stage of single‐shot learning.

In this study, the models were implemented using the PyTorch deep learning framework. All training was conducted on a Linux workstation equipped with an 80 GB NVIDIA A100 GPU.

### 2.6 State of the art methods

The proposed DFM is compared with other subspace reconstructions, with differences mainly in regularization.

#### 2.6.1 Method 1

A bilinear model generating spatial and temporal basis using DIP for dynamic MRI^34^ is

(8) $$\theta^{*}=\arg\underset{\theta }{\min}\|𝒜\left(G_{\theta }(U)V\right)-b{\|}^{2}$$

where the temporal basis V is precomputed from simulated signal evolution curves using the Bloch equation and a collection of realistic parameters. The input of CNN $G_{\theta }$ is a zero‐filled estimation of U. The optimal spatial basis is $G_{\theta^{*}}(U)$. The number of CNN layers in Method 1 follows DFM to ensure comparable implicit regularization from DIP. DFM includes 51714 trainable parameters. Method 1 requires 83040 trainable parameters. Note that even though the number of layers in CNN is the same for DFM and Method 1, the input and output channels of DFM are 16 and 2, respectively. Method 1 requires 32 channels of input and output corresponding to 16 spatial factors.

#### 2.6.2 Method 2

A conjugate gradient (CG), iterative sense reconstruction with a low‐rank representation using a known basis function as in Method 1 is described as

(9) $$U^{*}=\arg\underset{U}{\min}\|𝒜\left(UV\right)-b{\|}^{2}+\lambda \;ℛ(U),$$

ℛ denotes quadratic regularization, $ℛ(U)=\|U{\|}^{2}$, on the spatial basis with $\lambda =1\times 10^{-3}$.

#### 2.6.3 Method 3

Method 3 is similar to Method 2 described above, with the exception of wavelet spatial regularization on U. In particular, we choose $ℛ(U)=\|WU{\|}_{1}$, where W is the wavelet operator,^19^ with the $\lambda =2\times 10^{-4}$.

#### 2.6.4 Method 4

This method utilizes a multipass parameter fitting of zero‐filled images reconstructed with a subspace approach using known temporal basis functions, that is $U^{*}={𝒜}^{H}\left(bV^{H}\right)$ as described in.^46^ The general idea is to enforce the smoothness of the $B_{1}$ and inversion efficiency maps to prevent noise propagation into the $T_{1}$ maps. This is achieved by first estimating the four unknowns: $T_{1}$, $B_{1}$, inversion efficiency, and complex scaling factors. $B_{1}$ is denoised with a Gaussian filter and fixed as a known input for the next pass. The following three passes are to denoise the inversion efficiency map, being careful not to smooth across tissue types. The final $T_{1}$ maps are then lightly denoised with total variation minimization to improve SNR by about 5%.

To compare the quality of source images, only the first pass fitting is applied, and the additional smoothness regularization is disabled for Methods 1, 2, 3, and DFM. Methods are concluded in Table 1. DFM, Methods 1, 2, and 3 are iterative reconstruction methods to solve for the spatial basis. Method 4 obtains a spatial basis using gridding.

**TABLE 1:** Description of methods.

Method	Iterative reconstruction	Regularization	Regularized parameter fitting
DFM	✓	CNN	✘
Method 1	✓	CNN	✘
Method 2	✓	Quadratic	✘
Method 3	✓	Wavelet	✘
Method 4	✘	✘	✓

### 2.7 Evaluation metric

The quality of the source images is evaluated by the Peak signal‐to‐noise ratio (PSNR). PSNR is computed between a pair of images for a specific contrast. The accuracy of $T_{1}$ map is estimated by Normalized Root Mean Square Error (NRMSE).

(10) $$\text{NRMSE}(x,y)=\frac{\sqrt{\text{MSE}(x,y)}}{\text{Mean}(y)},$$

where x is the image to be evaluated based on the reference image y. Mean(y) denotes the average voxel intensity of the reference volume y.

## 3 RESULTS

### 3.1 Phantom study

**FIGURE 2:**
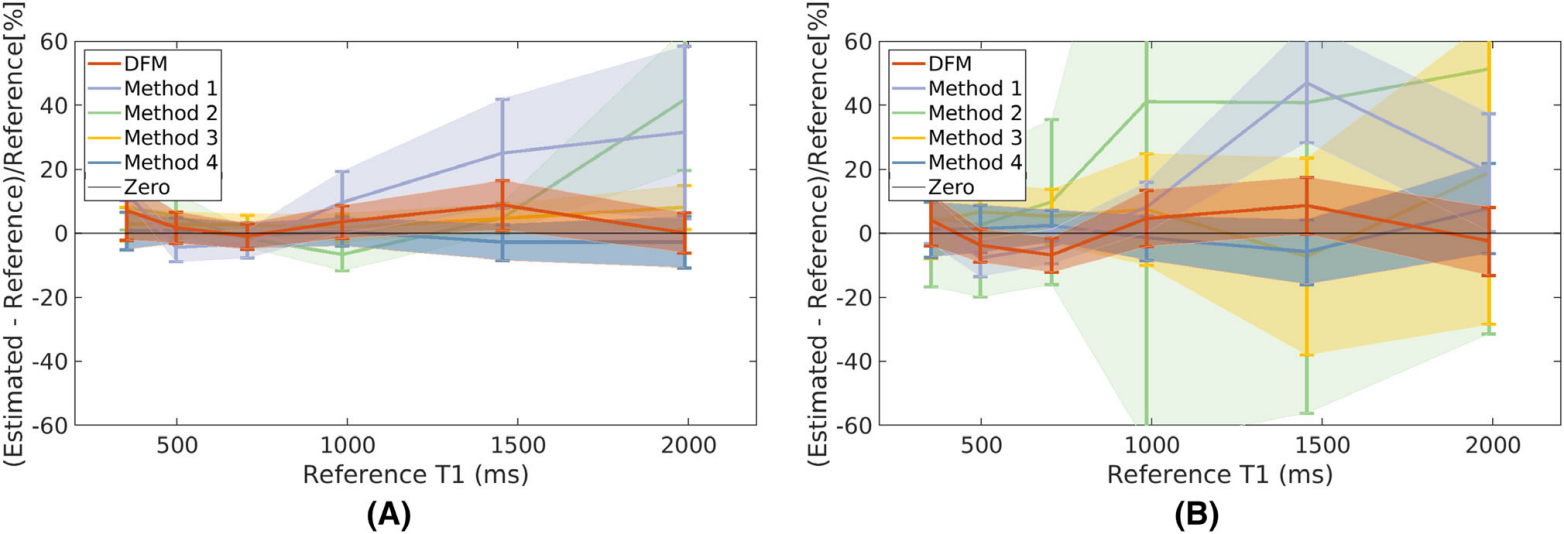
Plots of percentage errors compared to reference $T_{1}$ values. The X‐axis indicates the reference $T_{1}$ values. Comparisons are shown for $T_{1}$ estimations from DFM, Methods 1, 2, 3, and 4 for (A) 9 min; (B) 2.3 min acquisitions. The $T_{1}$ estimates from DFM exhibit lower bias and variability, maintaining high accuracy even at shorter scan times. In contrast, the other methods show increased bias and standard deviation. The $T_{1}$ estimates from DFM, obtained without spatial regularization, are comparable to those from Method 4.

The results from the phantom undersampling experiment for quantitative $T_{1}$ estimation are shown in Figure 2. Figures S1 and S2 in Supporting Information show quantitative $T_{1}$ maps and multicontrast images from 9, 4.5, 2.3, and 1.1 min scans. Figure 2 shows that the error and standard deviation of $T_{1}$ estimates for DFM remain highly accurate even at shorter scan times, while those of the other methods exhibit increased bias and variability. The $T_{1}$ estimates from DFM, obtained without spatial regularization, are comparable to those of Method 4.

### 3.2 In vivo study

#### 3.2.1 Single‐shot learning

**FIGURE 3:**
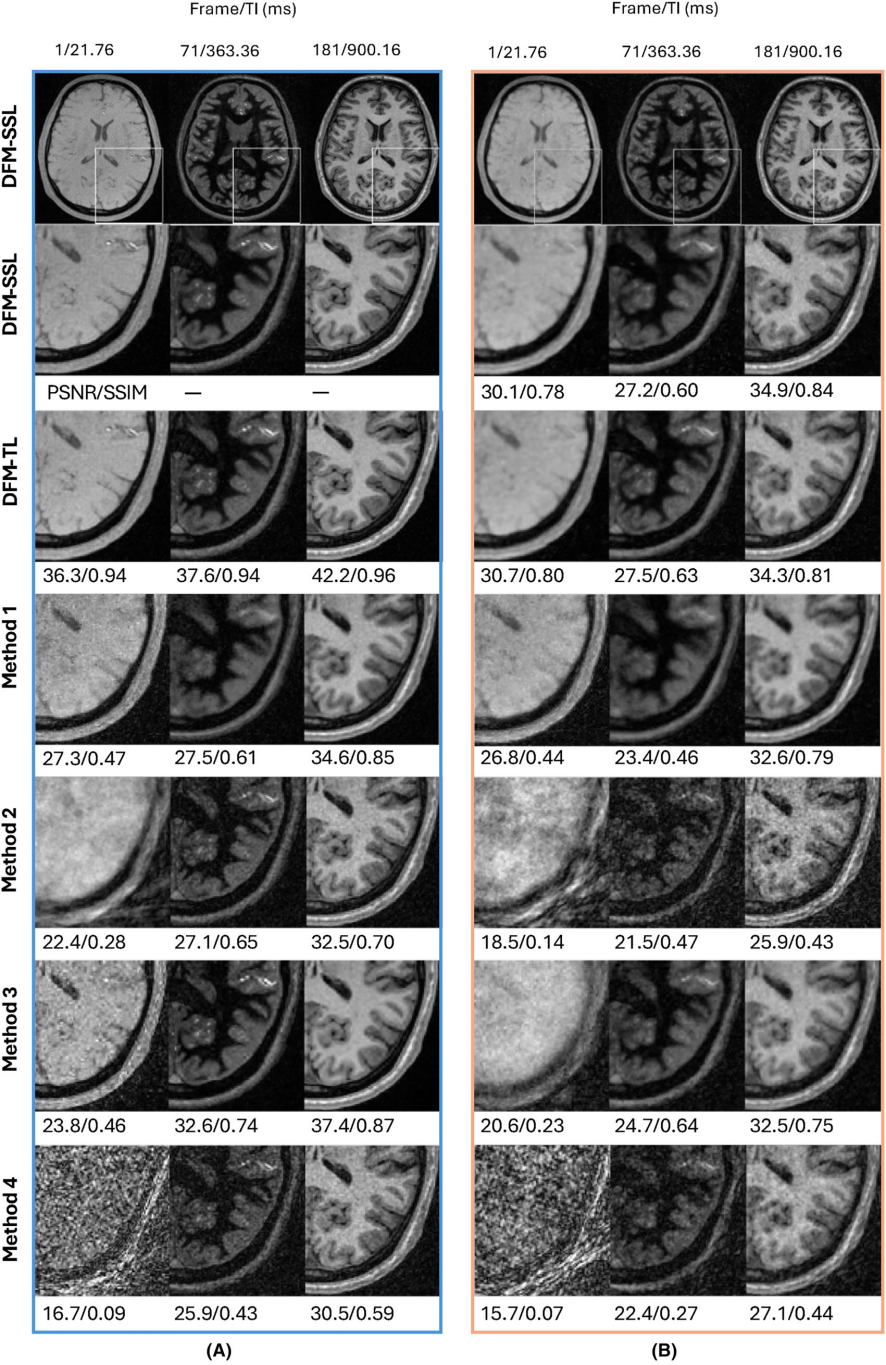
Comparison of methods at different accelerations. (A) 9 min scan; (B) 2.3 min scan; DFM‐SSL offers multicontrast images with less noise compared to Methods 2 and 3 while producing sharper edges than Method 1. DFM‐SSL recovers images with reduced artifacts and sharper edges across TIs. DFM‐SSL maintains image quality even under accelerated acquisitions with a scan time of 2.3 min. DFM‐TL is comparable to DFM‐SSL and outperforms Method 1 for both 9 and 2.3 min scans.

The comparison of DFM‐SSL with state of the art (SOTA) methods is shown in Figure 3 in axial view. Image reconstruction for shorter inversion times is particularly challenging due to rapid signal variations. Subspace reconstructions with quadratic regularization, indicated by Method 2, exhibit noise amplification and aliasing artifacts for shorter TIs, for example, TI = 21.76 ms. The spatial wavelet regularization (Method 3) mitigates noise amplification; however, undersampling artifacts persist at certain contrasts (e.g., TI = 21.76 and 363.36 ms). Method 1 further reduces artifacts across contrasts for both 9 and 2.3 min scans, but results in edge blurring and loss of fine details. In comparison, DFM‐SSL recovers images with fewer artifacts and sharper edges across TIs. DFM‐SSL maintains image quality even under accelerated acquisitions with a scan time of 2.3 min.

The $T_{1}$ map from DFM‐SSL appears less noisy than both Method 2 and 3, implying a lower estimation variance. Method 1, as a deep learning‐based method, also offers lower variance, but the bias is higher than DFM‐SSL evidenced by NRMSE, except in the cerebrospinal fluid (CSF) region where the $T_{1}$ is outside the range that the sequence is optimized for. The accuracy of the $T_{1}$ map aligns with the degradation of the source images, as shown in Figure 3. $T_{1}$ maps from DFM‐SSL are comparable to those from Method 4, which uses a spatially regularized parameter estimation. The evaluation of white matter and gray matter across 6 subjects is shown in Supporting Information Figure S6.

#### 3.2.2 Reducing the computational burden of DFM using transfer learning

**FIGURE 4:**
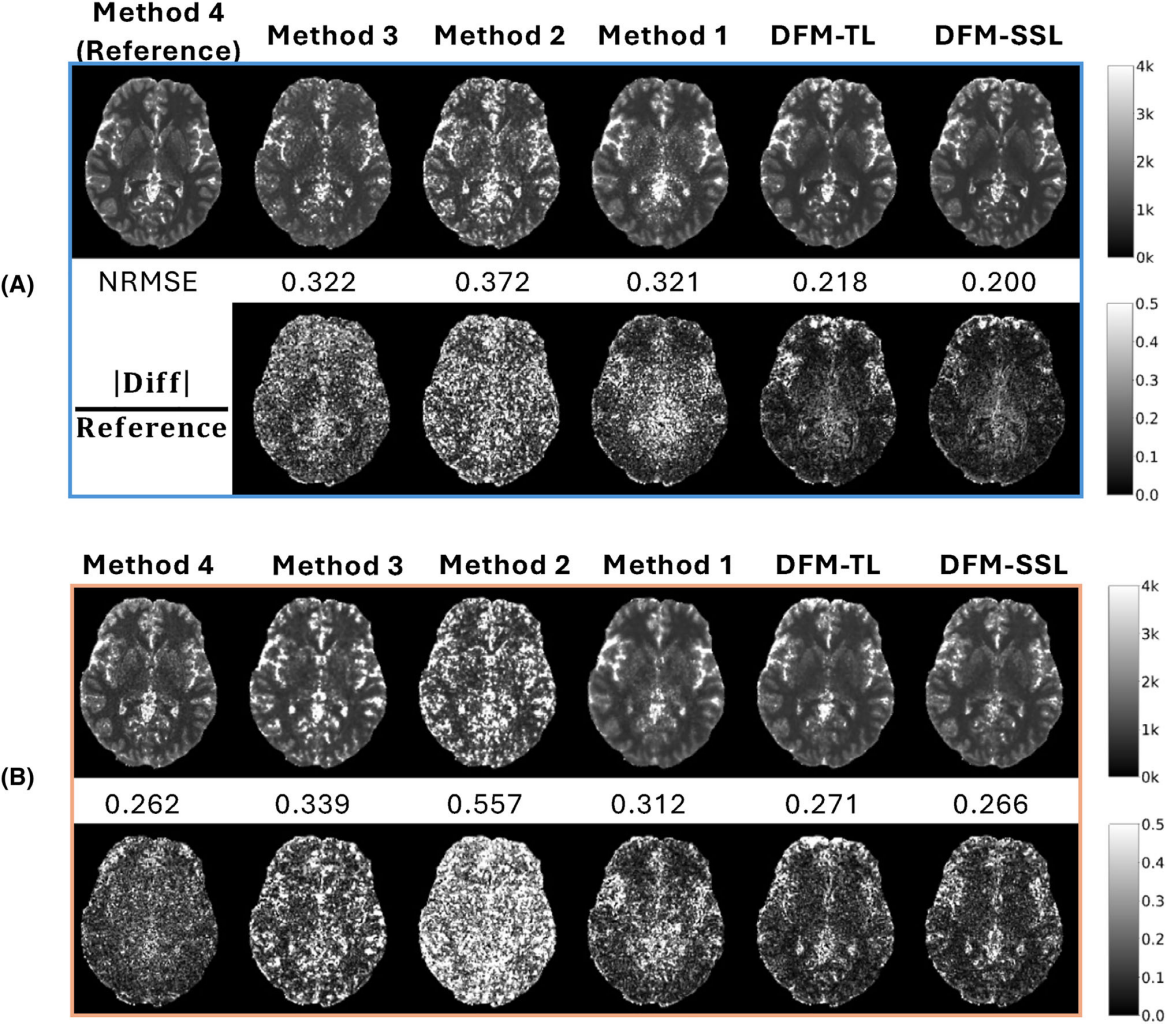
$T_{1}$ maps comparison. The reference $T_{1}$ map is from Method 4 with a 9 min scan. $\frac{\left|\text{Diff}\right|}{\text{Reference}}$ denotes $\left|\text{Reference}-\text{estimated}\;T_{1}\;\text{map}\right|$, normalized by Reference. The $T_{1}$ map from DFM‐SSL appears less noisy than both Methods 2 and 3, implying a lower variance of the estimation. Method 1, as a deep learning‐based method, also offers lower variance, but the bias is higher than DFM‐SSL, as evidenced by NRMSE, except in the cerebrospinal fluid (CSF) region where a $T_{1}$ is outside the range for which the sequence is optimized. (A) 9 min scan; (B) 2.3 min scan.

As shown in Figures 3 and 4, reconstructions from DFM‐TL achieve performance comparable to DFM‐SSL and outperform SOTA methods in both image quality and $T_{1}$ accuracy. Moreover, the transfer learning strategy significantly reduces the running time of DFM‐TL to 4.2 h from 18.9 h of DFM‐SSL for the 9 min scan. The reconstruction time and memory usage of other methods and undersampled scans are shown in Supporting Information Table S1. The average PSNR and SSIM across 384 contrasts, as shown in Supporting Information Table S2, also demonstrate the similar performance of DFM‐SSL and DFM‐TL.

## 4 DISCUSSION

In this work, we introduced a self‐supervised approach for the recovery of 3D radial multi‐contrast MPnRAGE data. The proposed deep factor model (DFM) generalizes linear factor models, including low‐rank methods, and offers implicit spatial regularization. The comparison of the proposed approach shows improved performance over traditional subspace methods over a range of scan times. In particular, the DFM approach shows the feasibility of recovering 384 inversion times with 1 mm isotropic resolution in around 2.3 min of scan time. The transfer learning strategy offers reduced inference time without compromising image quality.

Unlike unrolled deep learning algorithms^22^ that need multiple copies of coefficient images at each unrolling step, the proposed DFM scheme is significantly more memory efficient and therefore is well suited for the joint estimation of signal at the hundreds of time points available from the acquired MPnRAGE^4^ 3D inversion recovery raw data.

The main challenge with the proposed scheme is the long reconstruction time. Unlike supervised deep learning models, learning the network parameters from the measured k‐space data is computationally expensive. The supervised approach is not feasible in our setting, where extensive training data is unavailable. In addition, current unrolled models are associated with high memory demand during training, especially in the 3D multicontrast setting. Once multiple datasets are recovered using the proposed approach, these results may be used to train a supervised model, either in an end‐to‐end or plug‐and‐play fashion.

In this work, we have shown the feasibility of reducing the scan time using DFM. The proposed scheme can also be used to increase the spatial resolution of the dataset, which will be the focus of our future work. Similarly, the pulse sequence can be modified to include multiple quantitative parameters (e.g., $T_{2}$ in addition to $T_{1}$). Because it does not require training data, the proposed approach is well‐suited to these settings.

## DISCLOSURE

Curtis A. Corum is an employee of Champaign Imaging LLC.

## FUNDING INFORMATION

This work was supported by NIH grants: R01EB019961, R01AG067078, R01AG087159, R01EB031169, R01HD108868, R43MH122028, S10OD025025, P50HD103556, and P50 HD105353. The University of Iowa and University of Wisconsin‐Madison receive research support from GE Healthcare.

## Supporting information


**Data S1.** Supporting Information.
