# Supplementary material for: Accelerating 3D radial MPnRAGE using a self‐supervised deep factor model
Source: Magn Reson Med. 2025 Jun 2;94(3):1191–201. doi: 10.1002/mrm.30549 (PMC12202740; doi:10.1002/mrm.30549)
Supplement: Supplementary file 1 — Data S1. Supporting Information. [file MRM-94-1191-s001.pdf]

Supporting Information

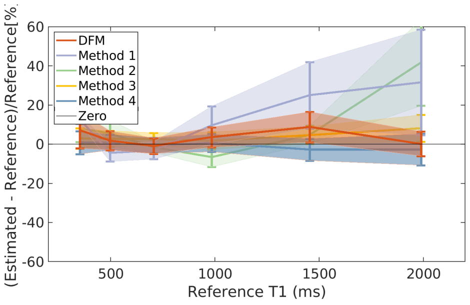

(a) 9 min

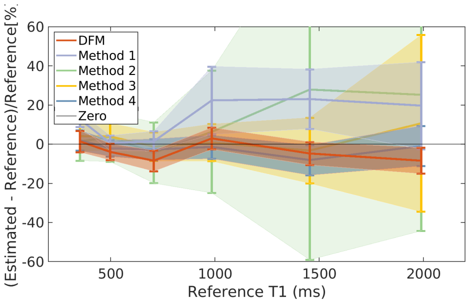

(b) 4.5 min

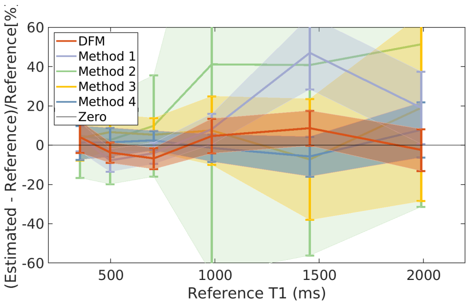

(c) 2.3 min

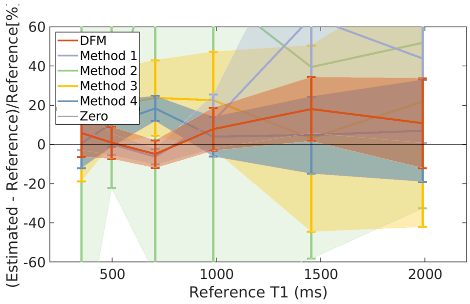

(d) 1.1 min

Figure S1: Plots of percentage errors compared to reference T_1 values. The X-axis indicates the reference T_1 values. Comparisons of T_1 estimations between DFM, Method 1, Method 2, Method 3, and Method 4 for acquisitions of (a) 9-min, (b) 4.5-min, (c) 2.3-min, and (d) 1.1-min. The 9-min scan with Method 4 serves as the reference. DFM shows minimal degradation up to 2.3-min acquisition.

	Memory usage(GB)				Runtime(hrs)			
Scan time(min)	9	4.5	2.3	1.1	9	4.5	2.3	1.1
DFM-SSL	74	72	68	66	18.9	9.7	7.5	5.8
DFM-TL	74	72	68	66	4.2	2.9	2.4	2.1
Method 1	80	75	71	69	4.6	2.9	1.9	1.4
Method 2	67	57	49	48	4.3 min	2.1 min	1.3 min	0.8 min
Method 3	67	57	49	48	2.6	2.2	2.1	2.2

Table S1: Memory and runtime comparison of different methods. Method 2 exhibits the fastest runtime among all methods, but it results in noisy source images and reduced T_1 accuracy. With CNN as a denoiser, the most time-consuming part of training is the multi-channel NUFFT forward operation, which depends on the k-space sampling rate. As a result, DFM and Method 1 achieve a significant reduction in runtime for accelerated scans. Method 1 runs faster than DFM-SSL but requires more memory. DFM-TL achieves a runtime comparable to Method 1 while maintaining image quality and T_1 accuracy comparable to DFM-SSL, as demonstrated by the phantom and in vivo study.

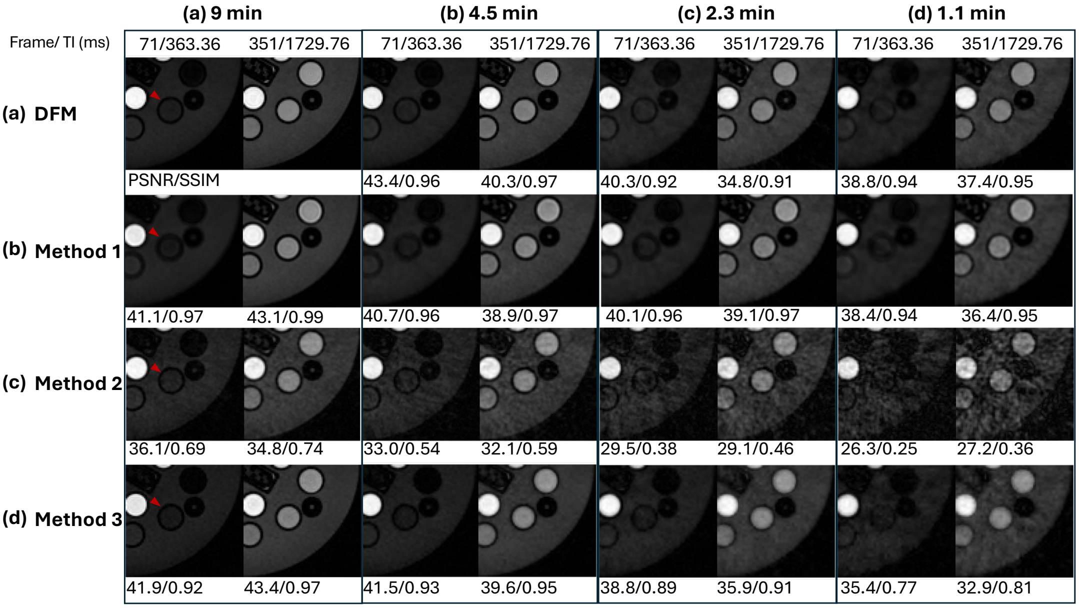

Figure S2: Phantom images comparison. We show two contrasts, corresponding to inversion times of 363.36 and 1729.76 ms, respectively. Reconstructions from (a) 9-min scan; (b) 4.5-min scan; (c) 2.3-min scan; (d) 1.1-min scan, are shown. The PSNR and SSIM are computed between each reconstruction and the DFM 9-min reference. Method 3 has fewer aliasing artifacts than Method 2 but introduces more spatial blurring than DFM. Method 1 reduces aliasing artifacts compared to Methods 2 and 3, but the bias in estimated T_1 values is higher than DFM implying less accurate signal recovery.

Scan time(min)	9	4.5	2.3	1.1
DFM-SSL	PSNR/SSIM	35.0/0.85	32.6/0.78	30.8/0.76
DFM-TL	42.2/0.96	35.1/0.84	32.6/0.79	31.3/0.78
Method 1	31.7/0.78	30.3/0.74	29.8/0.73	28.5/0.72
Method 2	29.7/0.57	27.0/0.44	24.3/0.32	21.7/0.23
Method 3	35.3/0.84	31.7/0.72	28.6/0.61	28.4/0.59
Method 4	27.1/0.45	25.5/0.38	24.1/0.32	23.1/0.29

Table S2: Comparison of PSNR and SSIM across different scan times. DFM-SSL from the 9-min scan is used as the reference to compute PSNR/SSIM. PSNR and SSIM represent the mean values across all image contrasts. Method 1 with CNN as a denoiser outperforms Method 3 for highly accelerated scans, such as 2.3-min and 1.1-min scans. Among all methods, DFM-SSL achieves the highest PSNR and SSIM for accelerated scans. DFM-TL demonstrates performance comparable to DFM-SSL, as indicated by similar PSNR and SSIM values.

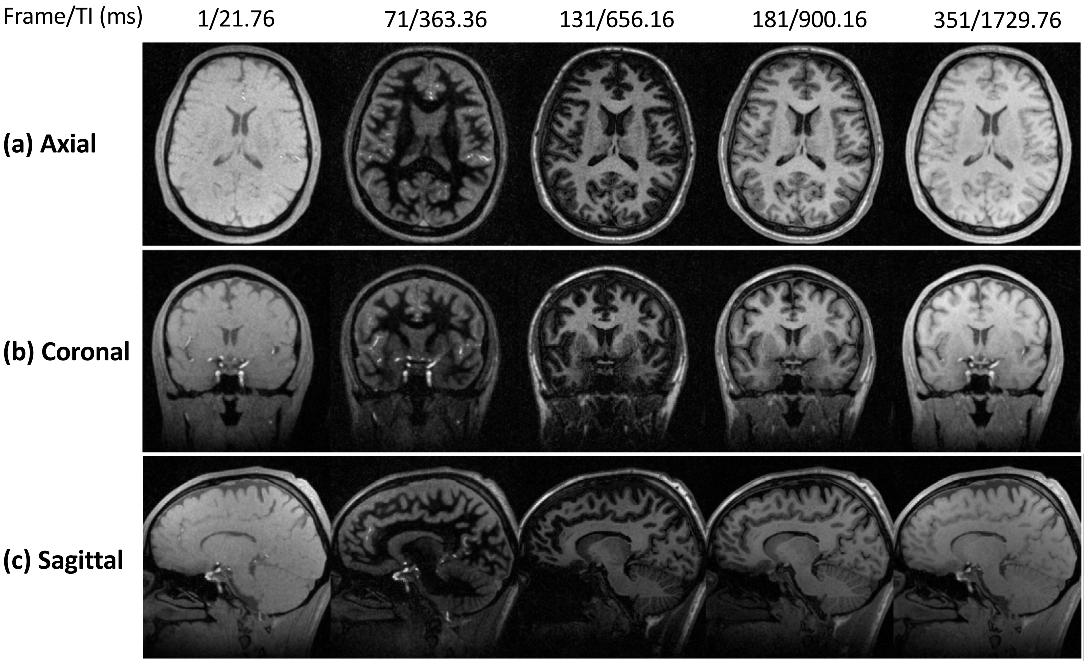

Figure S3: In vivo 3D DFM reconstructions from a 9-min scan. Axial, coronal, and sagittal views (shown as rows) are presented for five representative TIs chosen from the full inversion time-course. We note that the proposed DFM reconstruction is free from noise artifacts and offers clear differentiation between gray matter and white matter. DFM provides 3D multi-contrast images from the 9-min scan with visually reduced aliasing artifacts, which are consistent across contrasts and views.

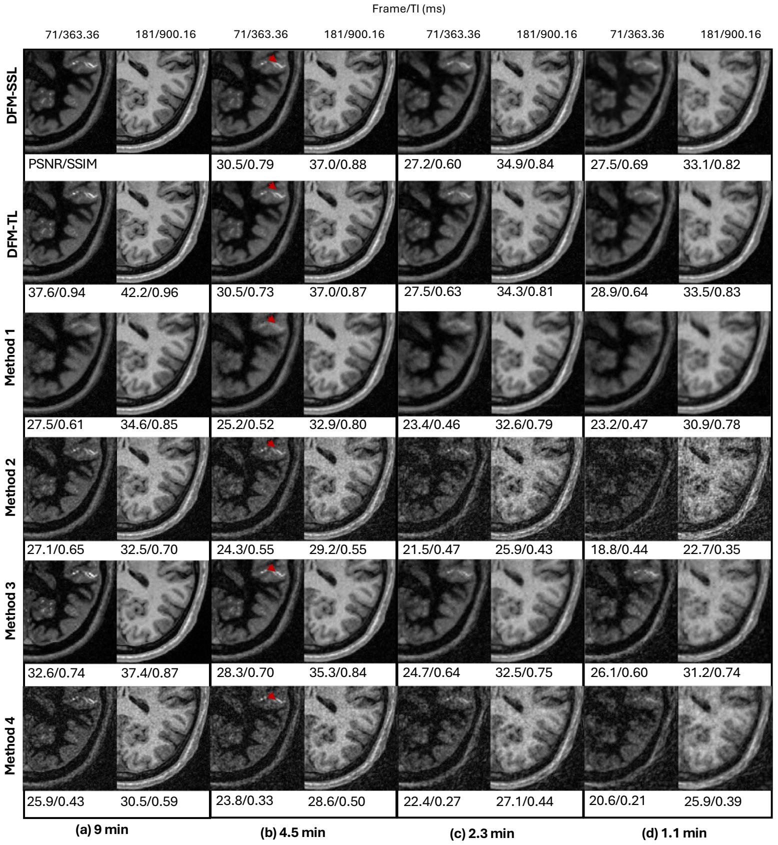

Figure S4: In vivo reconstructions at different accelerations. (a) 9-min scan; (b) 4.5-min scan; (c) 2.3-min scan; (d) 1.1-min scan. We report PSNR and SSIM using the DFM-SSL reconstruction from the 9-min scan as the reference. We observe that DFM-SSL offers multi-contrast images with less noise compared to Methods 2 and 3, while providing sharper edges than Method 1. DFM-TL is comparable to DFM-SSL both qualitatively and quantitatively.

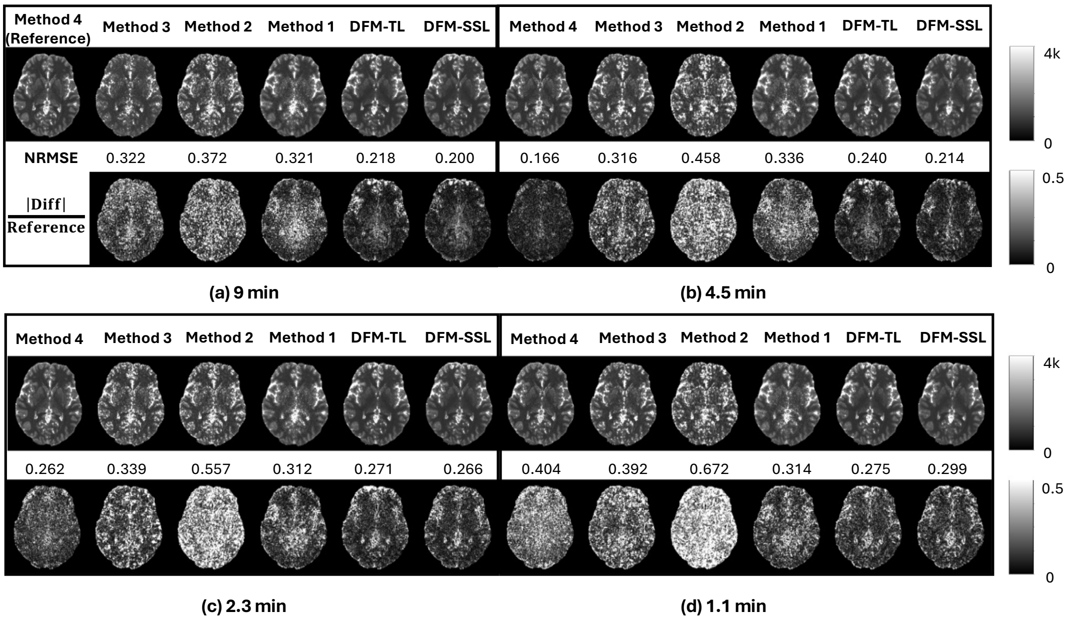

Figure S5: T_1 maps from multiple acceleration factors. (a) 9-min scan; (b) 4.5-min scan; (c) 2.3-min scan; (d) 1.1-min scan. We report NRMSE using Method 4 with the 9-min scan as the reference. The T_1 map from DFM-SSL shows a lower variance compared to Methods 2 and 3. Method 1 also provides lower variance, but suffers from higher bias, as indicated by higher NRMSE. DFM-SSL outperforms Method 4 at the 1.1-min scan.

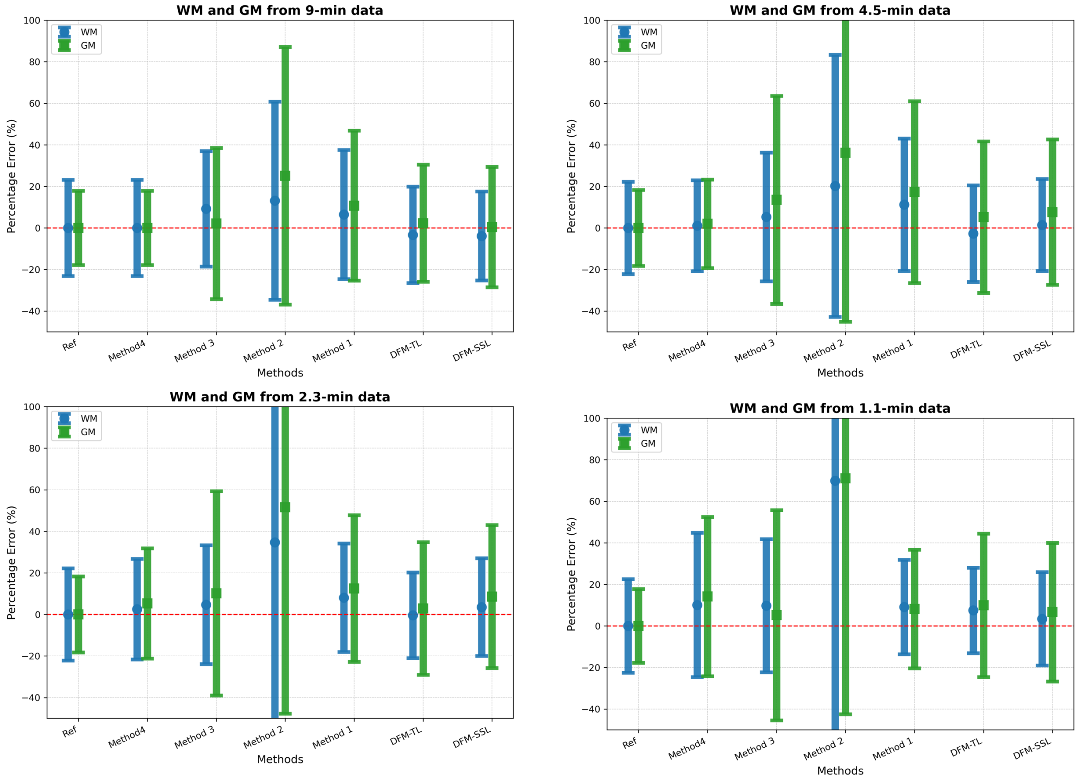

Figure S6: We evaluate the T_1 values of WM and GM from six in vivo subjects. For each subject, we segment the composite gridding reconstruction from the 9-min scan. The segmentation mask is then applied to all acceleration factors and methods to ensure a fair comparison of the source images. The reference (Ref) is obtained from Method 4 using the 9-min scan. We report the mean percentage error between the estimated values and the reference across the six subjects. As the acquisition time decreases, DFM-SSL exhibits a more gradual degradation, which aligns with the findings from the phantom study.

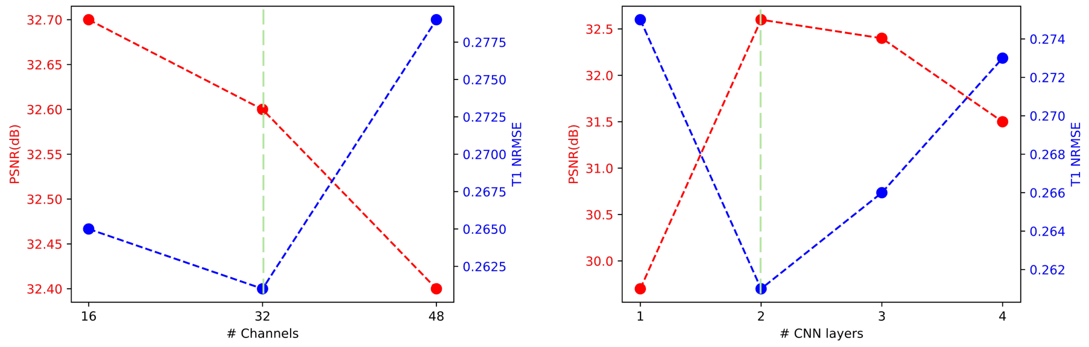

(a) PSNR and NRMSE versus channels of CNN

(b) PSNR and NRMSE versus layers of CNN

Figure S7: An ablation study on a 2.3-min scan, using DFM-SSL reconstruction from the 9-min scan as the reference. The T_1 map reference is obtained using Method 4 with the 9-min scan. Note that the number of layers in the CNN excludes the output layers. (a) The number of channels (features) in each layer of CNN: We fix the number of CNN layers at 2 and vary the number of channels. PSNR denotes the average over all image contrasts. We observe that 48 channels provide slightly higher PSNR than 32 channels but the NRMSE of T_1 is lower than 32 channels. (b) We study the effect of varying the number of CNN layers while keeping 32 channels of features. A 2-layer CNN provides the best performance in terms of both image quality and T_1 estimation.

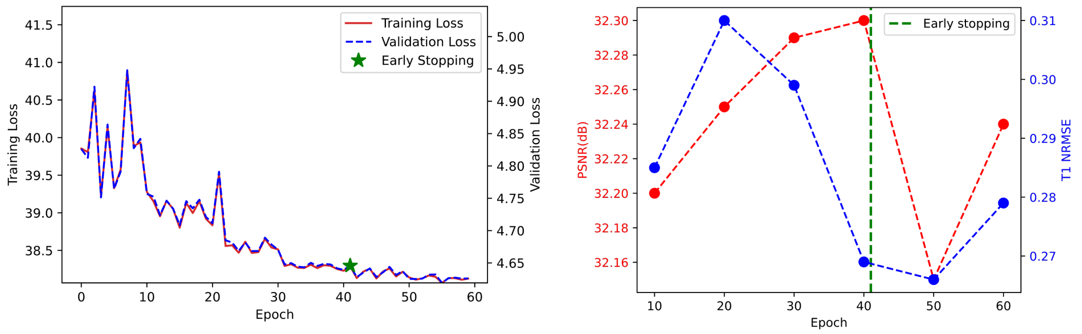

(a) Training and validation loss

(b) PSNR of multi-contrast images and NRMSE of T1 map versus epochs

Figure S8: Training with early stopping. We apply an early stopping strategy to prevent overfitting. We split 10% of the k-space data as validation data and use the remaining 90% for training. The data is split in a block-wise manner. The initial learning rate is 0.0005 and decreases by a factor of 0.2 if the validation loss does not decrease for 5 consecutive epochs. Training stops if the validation loss does not decrease for 10 consecutive epochs. (a) shows that training stops at epoch 41. (b) presents the PSNR and NRMSE every 10 epochs. This suggests that the optimal training performance is achieved around epoch 40.

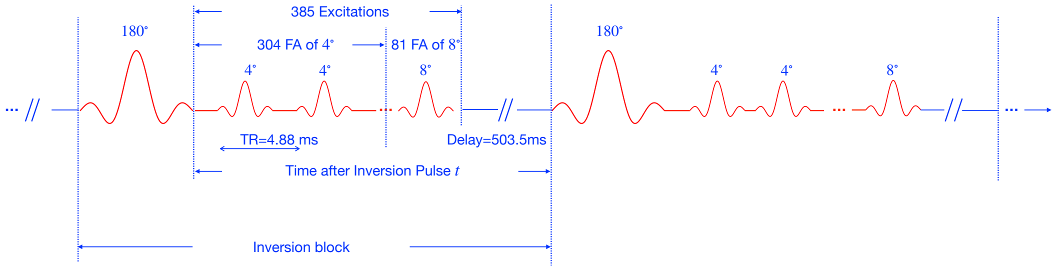

Figure S9: Pulse sequence parameters. The MPnRAGE scan was performed with FOV $= 256 \times 256 \times 256$ mm^3, resolution $= 1 \times 1 \times 1$ mm^3, TR = 4.88 ms, the duration of the inversion pulse = 12 ms. 385 radial readouts per inversion block were collected. The first 304 gradient echoes were acquired using a 4° flip angle, and the last 81 RF pulses were acquired with 8°. After the gradient echoes, a delay time of $T_D = 503.5$ ms allows the longitudinal magnetization to freely regrow before the next inversion pulse. 224 inversion blocks were acquired in 9 min. The dataset was retrospectively subsampled by only retaining a subset of inversion blocks. For example, retaining the first 56 inversion blocks corresponds to a 2.3-min scan.
